# Changes in the pattern of suicide attempters visiting the emergency room after COVID-19 pandemic: an observational cross sectional study

**DOI:** 10.1186/s12888-021-03570-y

**Published:** 2021-11-15

**Authors:** Ji-Hun Kang, Si-Won Lee, Jae-Gu Ji, Jae-Kwang Yu, Yun-Deok Jang, Seong-Ju Kim, Yang-Weon Kim

**Affiliations:** 1 Department of Emergency Medicine, Inje University Busan Paik Hospital, 75 Bokji-ro, Busanjin-gu, Busan, 47392 South Korea; 2 Department of Emergency Medical Technician, Dong Ju College. 16, Sari-ro 55beon-gil, Saha-gu, Busan, Republic of South Korea

**Keywords:** Attempted suicide, COVID-19, Emergency room, Suicide

## Abstract

**Background:**

This study aimed to find out the change in the rate and pattern of suicide attempts during severe acute respiratory syndrome COVID-19 pandemic period.

**Methods:**

This study was a retrospective analysis of data collected as a part of an emergency room-based post-suicide management program. The data were collected through interviews and from medical records of suicide attempts, maintained in the emergency room, from January 19 to October 31, 2020, during the “COVID-19 period,” and those who attempted suicide from January 19 to October 31, 2019 “pre-COVID-19 period.” We extracted educational background, marital status, occupation, presence of domestic partner, history of mental illness, alcohol consumption, history of previous suicide attempts; suicide attempt method and location (i.e., at home or a place other than home) at the time of attempt, and whether the attempt was a mass suicide. In addition, we compared patient severity between “COVID-19 period” and “pre-COVID-19 period” using the initial KTAS (South Korean triage and acuity scale) level, consciousness level, and systolic blood pressure. In 2012, KTAS was developed through the Ministry of Health and Welfare’s research project to establish triage system in South Korea.

**Results:**

The analysis of the number of suicide attempts during “pre-COVID-19 period” and “ COVID-19 period” showed that the number of suicide attempts during “COVID-19 period” (*n* = 440) increased compared to the “pre-COVID-19 period” (*n* = 400). Moreover, the method of suicide attempts during “COVID-19 period” included overdose of drugs such as hypnotics, antipsychotics, and pesticides that were already possessed by the patient increased compared to the “pre-COVID-19 period” (*P* < 0.05). At the time of the visit to the emergency room, high KTAS level, low level of consciousness, and low systolic blood pressure, were observed, which were significantly different between “COVID-19 period” and “pre-COVID-19 period” (P < 0.05).

**Conclusion:**

With the worldwide COVID-19 virus spread, suicide rate and suicide attempts at home have significantly increased. In addition, patient severity was higher in the “COVID-19 period” than that in the “pre-COVID-19 period.” The increasing suicide attempt rate should be controlled by cooperation between the emergency room and regional organizations.

## Background

According to the World Health Organization (WHO), suicide is defined as a self-injurious behavior that results in fatal consequences to one’s own body [1]. This risk can also be determined from a series of actions called suicidality [2]. Every 40 s an individual attempts suicide worldwide (approximately 793,000 people). South Korea has a high number of suicide attempts and re-attempts, which have been increasing each year. This has a negative impact on the society and economy as well as national development [3]. South Korea is ranked first in suicide rate among the member countries of the Organization for Economic Cooperation and Development [4]. In the past, financial hardship due to the national recession was the primary reason for suicide attempts in the South Korea. Recently, there has been an increase in suicide attempts due to mental illness and other reasons including copycat suicides (e.g., via media among youths) [5]. Therefore, in South Korea, a cross-ministerial cooperation has been devised to prevent suicides and suicide attempts. In particular, the ‘Management Project for Individuals who Attempt Suicide for the Emergency Room’, works on preventing suicide attempts and re-attempts in individuals admitted to the emergency room after a suicide attempt in South Korea. The main purpose of this program is preventing suicide and re-attempts suicide after visit emergency room in South Korea. It also plays a role in linking resources of the local government community in Busan, South Korea through called the Crisis Response Centre. The center operates with the collaboration of the Department of Emergency Medicine, Department of Psychiatry, and case management team, which provide counseling and treatment and run mental health and welfare projects.

Despite these efforts, there are still concerns that symptoms, such as anxiety and depression, caused by refraining oneself from external contact and going out because of the fear of COVID-19, may lead to an increase in suicide rates [6]. In fact, Ghebreyesus et al. [7] argued that a global pandemic, such as COVID-19, deteriorates neuropsychiatric state, which increases depression and anxiety. Unützer et al. [8]. claimed that the spread of COVID-19 increases depression, thereby increasing suicide rates and promoting suicidal behavior among individuals. Therefore, the WHO urges people around the world living in the COVID-19 era to reinforce the mental healthcare system. This study aims to examine changes in the general characteristics and patterns of suicide attempts and re-attempts from individuals admitted to the emergency room after the COVID-19 pandemic compared to the pre-COVID-19 period. The findings from this study may help to inform policies aimed at reducing suicide rates to the ER. As such, we examine the available data on the change in suicide types after the spread of COVID-19.

## Methods

### Participants

**Fig. 1:**
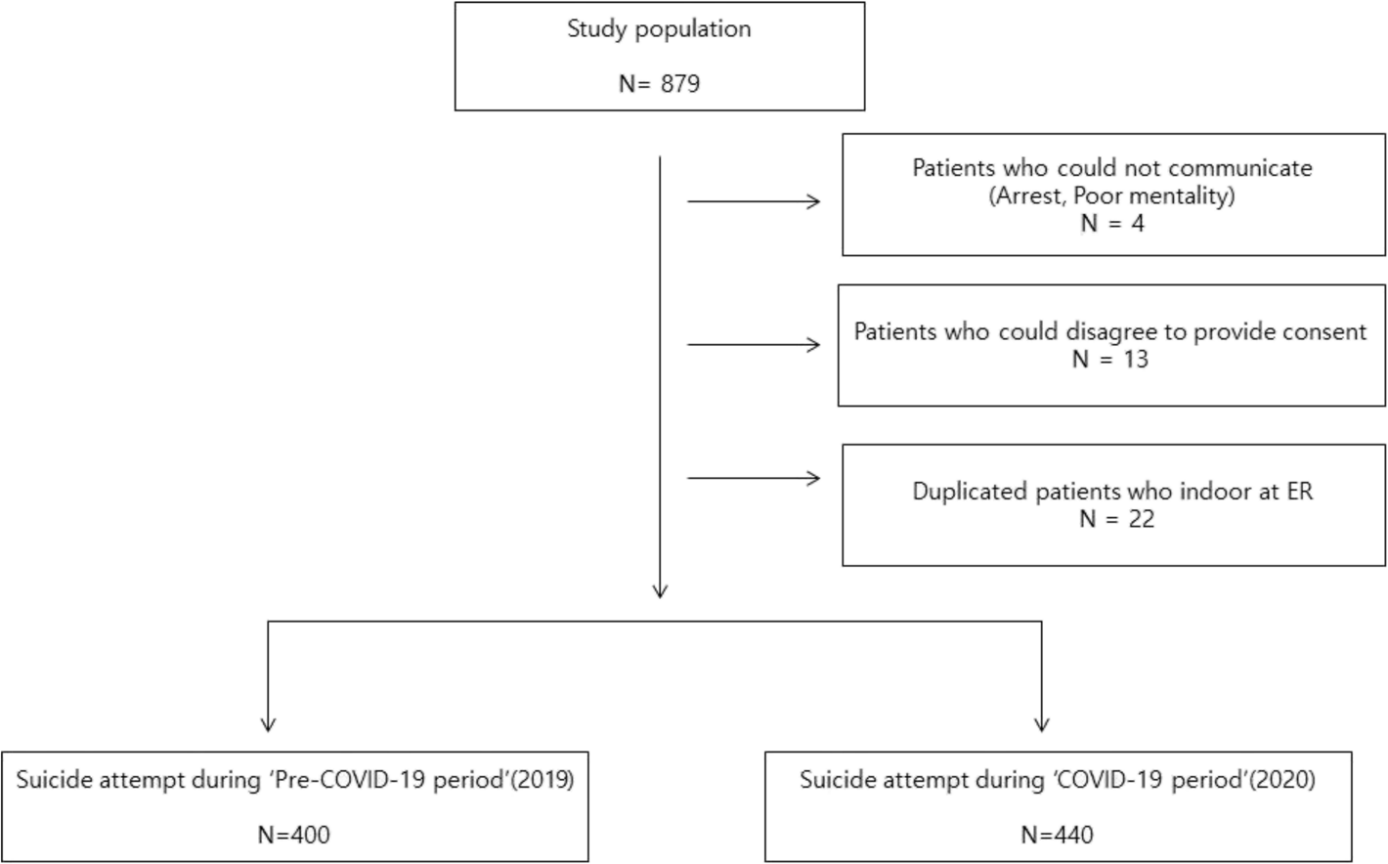
Study flow chart. ER Emergency room

For this study, a retrospective analysis was completed. Data was collected from the Busan University Hospital emergency room medical records. Busan is the second largest city in South Korea and the hospital sees about 30,000 patients annually. Interviews were conducted after obtaining verbal consent. And we also obtained written informed consents from all the participants prior to conducting the interview at the Crisis Response Centre. Participants included 879 individuals admitted to the emergency room during the COVID-19 period (January 19–October 31, 2020) and the pre-COVID-19 period (January 19–October 31, 2019) after a suicide attempt or re-attempt. Finally, the study included 840 participants after excluding 4 patients who had visited the emergency room without a guardian or who could not be interviewed (e.g., in situations of arrest, poor mentality, unable to communicate, etc.) and 13 patients who did not provide consent to the interview specialists to record data at the Crisis Response Centre. We also excluded the same individuals 22 suicide attempters who had more than two re-attempts (Fig. 1).

### Data collection and analysis

All study procedures comply with relevant national and institutional guidelines and regulations on care and clinical research standards. All data were encrypted, and the study protocol was approved by the Inje University Busan Paik Hospital in Busan, South of Korea (Institutional committee name, IRB No: 2020–11,002-003).

In order to collect data from electronic medical records, we extracted basic information, including gender, age, vital signs, level of consciousness at the time of visit (Alert = 1, verbally responsive = 2, pain responsive = 3, unresponsive = 4), and South Korean triage and acuity scale (KTAS) at the time of visit to the emergency room. As for the interview data, we requested compiled interview results of patients admitted to the emergency room after suicide attempt and re-attempt from the “Crisis Response Centre”, which contain the following information: educational background, marital status, occupation, presence of domestic partner, history of mental illness, alcohol consumption; and suicide attempt, suicide method, and location (i.e., at home or a place other than home) at the time of attempt, and whether the attempt was a mass suicide. We analyzed whether suicide patterns changed after the spread of COVID-19. In addition, in order to determine whether there was a change in severity, we collected and compared data between the COVID-19 and pre-COVID-19 groups regarding KTAS grade, level of consciousness, and systolic blood pressure at the time of admission to the emergency room. KTAS is common triage system in South Korea. The KTAS consists of five acuity levels; from level 1 to level 5. In the case of level 1, medical treatment by a specialist is required within 30 min after severity classification, level 2 requires medical treatment within 60 min, and level 3 requires medical treatment within 3 h. Screening with the KTAS occurs first with the serious and life-threatening conditions (for example, cardiac arrest, mental change of 8 points or less in Glasgow coma scale, and shock status) among patients, with a critical first look. In most patients who are not in a very critical condition, the KTAS assessment starts with the main complaint of the patient; the KTAS then takes into account additional modifiers such as the vital signs, the level of consciousness, pain severity, injury mechanism, and blood sugar. Using the SAS 9.3 software, the collected data were analyzed to identify general characteristics of the patients and express them in terms of frequency and percentage. In order to investigate the change in patterns of suicide attempts after the spread of COVID-19 (COVID-19 period; January 19–October 31, 2020), we performed descriptive statistics and average differences analyses. In addition, in order to examine changes in the number, method, and location of suicide attempts, we conducted a cross-analysis between the COVID-19 period (January 19–October 31, 2020) and the pre-COVID-19 period (January 19–October 31, 2019). An independent sample t-test was used to compare the change in severity among individuals admitted to the emergency room after suicide attempt during the “pre-COVID-19 period” and the “COVID-19 period.”

## Results

### General characteristics

The general characteristics of patients who visited the emergency room following a suicide attempt during the “pre-COVID-19” and “COVID-19” periods are shown in Table 1.

**Table 1:** Demographic statistics of “pre-COVID-19 period” and “COVID-19 period”

Variables		pre-COVID-19 period (***N*** = 400)		COVID-19 period (***N*** = 440)	
		N	%	N	%
Age (Mean ± SD)		40.68 ± 19.32		40.90 ± 19.66	
Sex	Male	150	37.5	157	35.70
	Female	250	62.5	283	64.30
Marriage	Single	179	44.8	196	44.5
	Married	114	28.5	123	28.0
	Partner	17	4.3	26	5.9
	Separated	30	7.5	30	6.8
	Divorced	45	11.3	44	10.0
	Widowed	15	3.8	21	4.8
Occupation	Executives	1	0.3	45	10.2
	Professional/technical	5	1.3	7	1.6
	Office work	51	13.1	18	4.1
	Service/retail	94	23.5	43	9.8
	Agriculture/fishing	2	0.5	0	0
	Skilled/technical	13	3.3	13	3.0
	Simple labor	12	3.0	23	5.2
	Military	2	0.5	1	0.2
	Student	69	17.3	72	16.4
	Homemaker	40	10.0	47	10.7
	Unemployed	111	27.8	171	38.9
Last stage of education	Never schooled	36	9.0	76	17.3
	Elementary school	51	12.8	35	8.0
	Middle school	65	16.3	90	20.5
	High school	166	41.5	179	40.7
	Undergraduate	82	20.5	60	13.6
Mass suicide (Partner status)	Yes	258	64.5	265	60.2
	No	142	35.5	175	39.8
Psychiatric history	Yes	163	40.8	381	86.6
	No	237	59.3	59	13.4
Alcohol consumption history	Yes	186	46.5	208	47.3
	No	214	53.5	232	52.7

N number of participants with that characteristic, SD standard deviation

Data were presented as number (%) or mean and standard deviation were used in Table

And we conducted analysis except in cases where the patient repeatedly visited the emergency room to prevent possible inflation of cases. The number of patients who visited the emergency room following a suicide attempt was 400 during the “pre-COVID-19 period” and 440 during the “COVID-19 period.” There were more women than men and more unmarried than married patients who visited the emergency room following a suicide attempt during both periods. A comparison between the two periods regarding occupation showed that there was only one case of an executive (0.3%) who attempted suicide during the “pre-COVID-19 period,” whereas the number increased to 45 (10.2%) during the “COVID-19 period.” The number of patients who had a history of psychiatric treatment was 163 (40.8%) during the “pre-COVID-19 period” and 381 (86.6%) during the “COVID-19 period.” The number of double suicide attempts was 379 during “COVID-19 period,” which was higher than that during the “pre-COVID-19 period” (Table 1).

### Method and location of suicide attempts

Table 2 shows the difference between the location and method of suicide attempts during the “pre-COVID-19 period” and the “COVID-19 period.” The number of suicide attempts at home was higher during the “COVID-19 period” (403, 91.6%) than the “pre-COVID-19 period” (364, 90.9%; *P* < .05). When we analyzed the method of suicide attempts, the number of suicide attempts by overdosing prescribed antipsychotics or sleeping pills was higher during the “COVID-19 period” (251, 66.6%) than the “pre-COVID-19 period” (48, 14.4%; *P* < .05) (Table 2).

**Table 2:** Change in location and method of suicide between “pre-COVID-19 period” and “COVID-19 period”

variable		pre-COVID-19period (*N* = 400)		COVID-19 period (*N* = 440)		*χ*_2_	*P*-value
		N	%	N	%		
Suicide location	home	364	90.9	403	91.6	263.402	.000
	place other than home	36	9.1	37	8.4		
Suicide method	drug overdose	48	13.3	251	66.6	223.992	.000
	self-harm injury	299	82.6	113	30.0		
	gas poisoning	6	1.7	3	0.8		
	fall	1	0.3	0	0.0		
	hanging	6	1.7	7	1.9		
	drowning	1	0.3	1	0.3		

COVID-19 Coronavirus disease 2019, N number. Chi-square tests, were used in Table 2. and *P* < 0.05 was deemed statistically significant

### Change in severity of patients who attempted suicide

Table 3 shows the change in severity of patients who visited the emergency room following a suicide attempt during the “pre-COVID-19” and “COVID-19” periods. The KTAS grade at the time of admission was higher during the “COVID-19 period” (1.10 ± 0.33) than the “pre-COVID-19 period” (2.01 ± 0.23; *P* < .05).

**Table 3:** Severity compared with between “pre-COVID-19 period” and “COVID-19 period”

Variation	pre-COVID-19 period (*N* = 400)			COVID-19 period (*N* = 440)			t	***p***-value
	Mean ± SD	Min	Max	Mean ± SD	Min	Max		
Initial KTAS level	2.01 ± 0.23	1	3	1.10 ± 0.33	1	2	44.819	.000
Mentality (AVPU)	1.52 ± 0.76	1	3	2.73 ± 0.80	1	4	22.323	.032
Systolic BP	116.72 ± 69.60	95	120	90.32 ± 35.85	80	110	21.579	.023

COVID-19 Coronavirus disease 2019, N number, KTAS South Korean triage and acuity scale, A alert, V verbal response, P pain response, U un-response, BP blood pressure, Min minimum, Max maximum, SD standard deviation. Student’s t tests were used in Table 3 and *P* < 0.05 was deemed statistically significant

The level of consciousness at the time of admission was lower during the “COVID-19 period” (2.73 ± 0.80) than during “pre-COVID-19 period” (1.52 ± 0.76; *P* < .05). The systolic blood pressure at the time of admission was lower during the “COVID-19 period” (90.32 ± 35.85) than during “pre-COVID-19 period” (116.72 ± 69.60; P < .05) (Table 3).

## Discussion

In this study, we found that suicide patterns have changed. During the “COVID-19 period”, the number of suicide attempts appearing int the emergency room increased and has been more severe compared to the pre-COVID-19 pandemic period examined. There are several reasons that may explain observed results. First, the increase in the number of emergency room members of “COVID-19 period” suicide attempts are considered to be due to the increased social fear and mood depression after the spread of the coronavirus. Secondly, those who were likely to visit the hospital due to suicidal ideations are now forced to stay home during the COVID-19 period unless their medical condition has deteriorated to a point where medical care becomes essential. This would cause an increase in severity of patients visiting the ER during the COVID-19 period.

Despite the South Korea government’s efforts to reduce suicide rates, the suicide rate to increased in Korea was 26.5 per 100,000 persons in 2015 [9]. In order to prevent consequent social and economic loss, it is important to establish cooperation between government organizations and ministries as well as develop preventive measures for the emergency room, which is the first place visited by most individuals who attempt suicide [10, 11]. Also, when comparing the “Pre-COVID-19 period” to “COVID-19 period”, it is clear that self-harm injury rates are significantly lowered. This may be due to the adherence of lockdown restrictions, where they would be forced to stay with their families and community. As such, this would lead to less emotional stress [12]. Hence, after assessing the risk of individuals who visit the emergency room following a suicide attempt, departments of emergency medicine and psychiatry cooperate on mental health support programs for post-discharge management [11]. Similarly, South Korea has also started the ‘Management Project for Individuals who Attempted Suicide for the Emergency Room’ in July 2013, in cooperation with the local governments, to prevent suicide re-attempts. However, it is difficult to provide continuous mental health support due to the current large-scale spread of COVID-19; the suicide rate is expected to increase due to the current social atmosphere.

In the past, respiratory virus infections have cause major upheavals around the world by disrupting infrastructure and global public health orders. The outbreak of Severe Acute Respiratory Syndrome (SARS), which began in Southeast Asia in March 2003 and spread all over the world, recorded 8906 cases, 774 deaths, and a mortality rate of 9.6%. In 2012, the death rate from the global outbreak of Middle East respiratory syndrome (MERS) reached up to 38% [13]. The outbreak of SARS COVID-19, which originated in Wuhan, China in December, 2020, is an ongoing pandemic with a death rate of 1–3% [14]. To prevent the spread of such respiratory infections around the world, various measures are being undertaken in each country. In particular, South Korea is trying to minimize contact between people by implementing measures, such as strengthening personal hygiene rules, social distancing between individuals, advising individuals to refrain from going outside, and closing schools and promoting online classes. However, these measures may worsen stress and depression in individuals with mental illness because of inability to support their mental health due to social isolation. In fact, Sher [6] argued that the threat of the pandemic increases mental stress as the fear of going outside and interpersonal contact increases. Furthermore, studies have shown that the pandemic affects the economic downturn and increases the number of unemployed people, which results in generating a new pool of individuals without a history of mental illness who might attempt suicide [15]. In fact, according to the data released by the International Labor Organization, nearly 25 million jobs have been lost worldwide due to the COVID-19 pandemic, based on which the WHO anticipated a 20-fold increase in the number of suicide attempts and recommended global mental health measures [15]. It is claimed that such large-scale spread of respiratory infections not only has an impact on social infrastructure and public health but also worsens symptoms, such as stress, depression, anxiety, and insomnia, with fear in the social atmosphere, thereby increasing the suicide rate [16]. Hence, given the already high suicide rate in South Korea prior to the pandemic, we expect that the large-scale spread of this infectious disease would inevitably increase the number of suicides and suicide attempts. As shown in the results of this study, the number of individuals who visited the hospital following a suicide attempt during the “COVID-19 period” (*n* = 440) was higher compared to that of the “pre-COVID-19 period” (*n* = 400). In particular, the rapid increase in the number of patients with a history of psychiatric treatment due to suicide attempts can be interpreted as a result of increased anxiety and depression caused by the lack of mental health treatment because of the current social environment and concerns about the infectious disease [6]. In addition, as shown in the results of comparison between suicide attempt locations, the number of suicide attempts at home increased rapidly during the “COVID-19 period” compared to the “pre-COVID-19 period,” as people spend more time at home to abide by the social distancing rule. The number of suicide attempts by overdosing prescribed psychiatric medications or sleeping pills was also higher during the “COVID-19 period” than the “pre-COVID-19 period.”

Hence, we suspect that the level of consciousness was lower during the “COVID-19 period” than the “pre-COVID-19 period,” whereas the KTAS grade was higher for patients who visited the hospital during the “COVID-19 period” than the “pre-COVID-19 period.” If the level of consciousness is low in individuals who attempt suicide by drug overdose, respiratory failure or aspiration pneumonia may interfere with ventilation and carbon dioxide may accumulate in the body, at which point the respiratory treatment becomes necessary. Moreover, this would increase the possibility of intensive care treatment as the severity and fatality of patients increase with a history of cardiac and respiratory diseases.

Our study showed that the rate of suicide attempts as well as the severity of patients who visited the hospital after a failed attempt increased due to the prolonged global threat of COVID-19 pandemic. In spite of the increase in the suicide rate and severity of patients who visit the hospital after a failed suicide attempt, South Korea lacks adequate measures to prevent suicides. On the other hand, other countries have already highlighted that the increase in suicide rate caused by the coronavirus may lead to social problems, and have recommended strengthening public mental health activities and policies [17]. Furthermore, countries are trying to prevent suicide attempts in advance by minimizing the anxiety and stress in patients with mental illness through cooperation between the local community and medical facilities while suggesting specific alternatives [18]. Our neighbor, China, has declared a national neuropsychological crisis, and has delegated specialists in each region, tracked down individuals for monitoring and management, and developed a guideline for their management, based on which patients at a high risk of suicide attempts are engaged in more active interviews. Additionally, they have used online education and media to disseminate the ‘Public Psychological Self-help Guideline for Pneumonitis with New Coronavirus Infection’ to educate people on how to reduce anxiety and stress [19]. Keeping pace with these global efforts, South Korea also needs to manage patients at a risk of suicide attempt at the emergency room level, develop online education methods that allow post-discharge interviews, and introduce educational programs that can help such individuals in reducing anxiety and stress by themselves at home.

This study has some limitations. First, it is difficult to consider that our study represents the entire population, because it is based on data from a single research institution, i.e., the emergency room-based sample data. Furthermore, given that the majority of patients at this institution consists of the elderly, our study does not reflect various age groups. Second, among the patient group that died in the intensive care unit, there can be other causes of death than the suicide attempt itself. Third, regional characteristics cannot be excluded from our study, which was conducted in a metropolitan city that had the highest suicide rate according to Statistics South Korea. Fourth, if this study included subjects that were analyzed more than once, this would significantly affect the results. Consequently, we excluded any patient ID numbers that were repeated during the analysis.

## Conclusion

The COVID-19 pandemic and resulting social unrest have increased anxiety and stress in people and thereby increased the suicide rate. Therefore, in order to prevent suicide attempts in advance, we need to establish cooperation between government organizations and local agencies and develop early and follow-up management of individuals who visit the emergency room after a suicide attempt.

## Data Availability

The datasets analyzed during the current study are available from the corresponding author on reasonable request.

## Abbreviations

COVID-19: Coronavirus disease 2019
KTAS: South Korean triage and acuity scale
SD: Standard deviation
SARS: Severe acute respiratory syndrome
AVPU: A = alert, V = verbal response, P = pain response, U = un-response
